# Over‐expression of mutated *ZmDA1* or *ZmDAR1* gene improves maize kernel yield by enhancing starch synthesis

**DOI:** 10.1111/pbi.12763

**Published:** 2017-07-25

**Authors:** Guangning Xie, Zhaoxia Li, Qijun Ran, Hui Wang, Juren Zhang

**Affiliations:** ^1^ School of Life Sciences Shandong University Jinan Shandong China

**Keywords:** maize, grain yield, ubiquitin receptor, transfer cell layer, starch content

## Abstract

Grain weight and grain number are important crop yield determinants. DA1 and DAR1 are the ubiquitin receptors that function as the negative regulators of cell proliferation during development in *Arabidopsis*. An arginine to lysine mutant at amino acid site 358 could lead to the *da1‐1* phenotype, which results in an increased organ size and larger seeds. In this study, the mutated *ZmDA1* (*Zmda1*) and mutated *ZmDAR1* (*Zmdar1*) driven by the maize ubiquitin promoter were separately introduced into maize elite inbred line DH4866. The grain yield of the transgenic plants was 15% greater than that of the wild‐type in 3 years of field trials due to improvements in the grain number, weight and starch content. Interestingly, the over‐expression of *Zmda1* and *Zmdar1* promoted kernel development, resulting in a more developed basal endosperm transfer cell layer (BETL) than WT and enhanced expression of starch synthase genes. This study suggests that the over‐expression of the mutated *ZmDA1* or *ZmDAR1* genes improves the sugar imports into the sink organ and starch synthesis in maize kernels.

## Introduction

Crop improvement is essential to meet the demands generated by explosive population growth and global climate warming. In recent decades, genetic engineering has been shown to represent a powerful means of improving grain yields in various crops, such as rice, wheat, and maize (Li *et al*., 2013). Improving the crop seed yield using unconventional means may significantly contribute to meeting the increasing global food demand.

Final seed size and weight are controlled by multiple factors, such as genetics, hormonal regulation and environmental impact (Xia *et al*., 2016). Several genes involved in cell proliferation, such as the *Cell Number Regulator 1* in maize and *Physalis floridana*, have been reported to contribute to crop production due to their function in cell number regulation during the seed development stage (Guo *et al*., 2010; Li and He, 2014). In monocots, endosperm size is the primary factor in kernel size determination. The seed size greatly depends on the endosperm cell number (Capitanio *et al*., 1983; Jones *et al*., 1996). A series of genes related to the endosperm development were recently identified. Some of the genes were reported to contribute to seed yield by controlling endosperm size (Luo *et al*., 2005; Schruff *et al*., 2006; Song *et al*., 2007), and the ubiquitin‐proteasome pathway has been reported to be important in controlling plant seed development (Disch *et al*., 2006; Du *et al*., 2014; Li *et al*., 2008; Peng *et al*., 2015; Xia *et al*., 2013).

In *Arabidopsis*, the ubiquitin‐proteasome system participates in cell number regulation (Li and Li, 2014), and the ubiquitin receptor DA1 is a negative regulator in seed size control. A single base mutant (*Atda1*) with an arginine to lysine change at the conserved amino acid position 358 (DA1R^358K^) leads to a *da1‐1* phenotype, and these mutants have larger leaves, flowers and seeds than the wild‐type (WT) (Li *et al*., 2008). AtDAR1 is the closest homologue of AtDA1 and has a molecular function that is redundant with AtDA1. Both AtDA1 and AtDAR1 have a negative effect on organ size control by restricting the period of cell proliferation in *Arabidopsis*. The T‐DNA insertion mutants *da1‐ko* and *dar1‐1* have not shown apparent phenotypes with respect to seed size; however, an increased seed size was observed in a *da1‐ko dar1‐1* double mutant (Li *et al*., 2008).

Proper enhancement of the import strength of the sink organ could improve grain crop yield. Previous studies showed that the over‐expression of genes such as glucose transporter, sucrose transporter or cell wall invertase in the ovule could improve seed yield (Li *et al*., 2013; Saalbach *et al*., 2014; Sosso *et al*., 2015). Both of the genes have an effect on the sugar import into the kernels and contribute to the development of endosperm.

In this study, we focus on the *ZmDA1* and *ZmDAR1* genes*,* which are the homologous genes of *AtDA1* and *AtDAR1* in maize. The *Atda1‐1* mutant has an increased seed size; thus, we postulated that the constitutive expression of *Zmda1* or *Zmdar1* would have a similar phenotype. The same single base mutant that leads to an arginine to lysine change at the conserved site was introduced into the *ZmDA1* and *ZmDAR1* nucleotide sequences. The two mutated genes were separately transformed into the maize inbred line DH4866. Both *Zmda1* and *Zmdar1* transgenic events showed an increased grain yield by enhancing the grain weight and grain number. The over‐expression of *Zmda1* and *Zmdar1* influenced the sugar import into the kernels. In the transgenic plants, more carbohydrate was imported into the kernels through a well‐developed endosperm transfer cell layer; this was linked to the increased starch content.

## Results

### Identification of the *ZmDA1* and *ZmDAR1* genes

The maize protein database was searched with BLASTP method using the AtDA1 and AtDAR1 amino acid sequences. ZmDA1 and ZmDARs belong to a sub‐family of the LIM gene family; there are 16 LIMs in maize. A total of 6 sequences were identified as members of the DA1 gene family, and both contain a single LIM domain, two UIM domains and an unknown function domain 3633 (DUF3633), which are the characteristic domains of the DA1 gene family. Most of the DA1 gene family members in maize and *Arabidopsis* have a similar expression pattern and are widely expressed in all stages and organs (Table S1).

**Figure 1:**
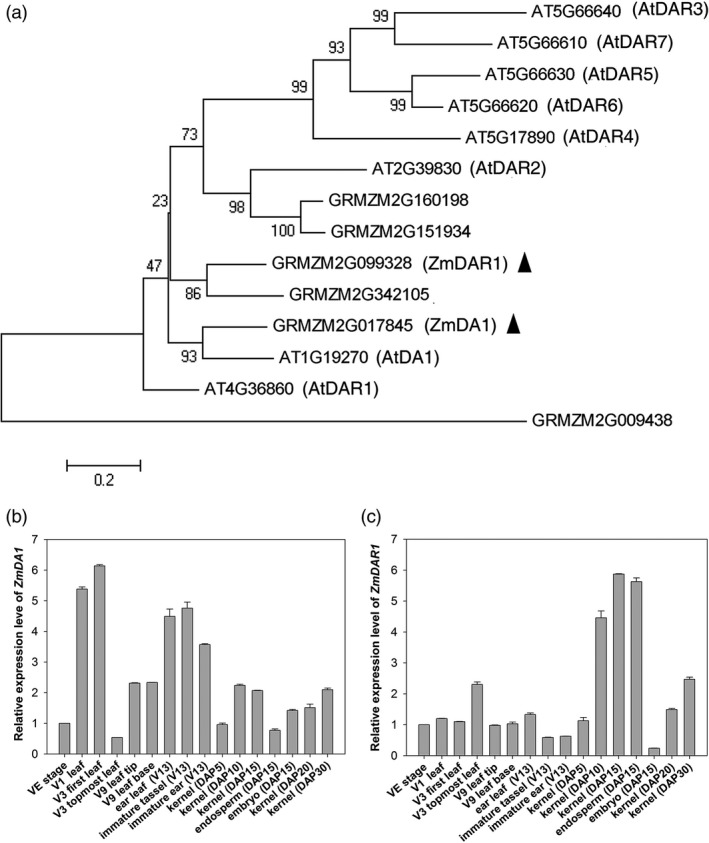
An evolutionary tree for the DA1 family, and the expression levels of the *ZmDA1* and *ZmDAR1* genes. (a) An evolutionary tree for the DA1 gene family in maize and *Arabidopsis*. (b, c) qRT‐PCR analyses of the *ZmDA1* and *ZmDAR1* gene expression patterns in different organs and stages in the maize inbred line DH4866. Values are the means ± SD;* n* = 6, three technical replicates and two biological replicates.

The amino acid sequences of AtDARs and ZmDARs were selected to build an evolutionary tree (Figure 1a). ZmDA1 and ZmDAR1 have the closest similarity with AtDA1 and AtDAR1 in terms of their amino acid sequences. *ZmDA1* encodes a 507‐amino acid protein, and *ZmDAR1* encodes a 503‐amino acid protein. Interestingly, AtDAR3 to AtDAR7 was a separate clustering in a clade in the evolutionary tree; however, ZmDA1 and ZmDARs had a closer relationship with AtDA1, AtDAR1 and AtDAR2. The expression patterns of *ZmDA1* and *ZmDAR1* were examined using qRT‐PCR. *ZmDA1* and *ZmDAR1* are ubiquitously expressed in maize plants. *ZmDA1* is primarily expressed in leaves and immature tassels and ears, while *ZmDAR1* is primarily expressed in kernels at DAP10 to DAP15; embryos show low expression at DAP15 (Figure 1b, c).

### Generation of transgenic plants

**Figure 2:**
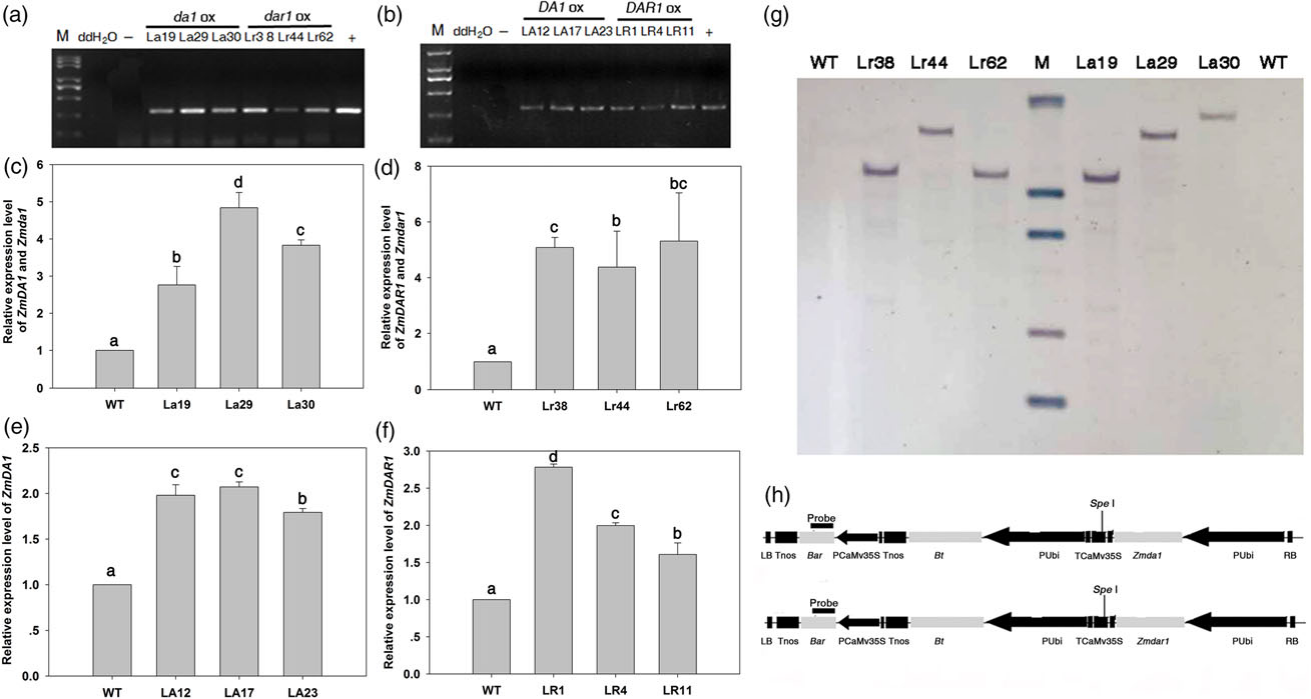
Molecular characterization of transgenic plants. (a and b) PCR assay for the bar gene in the transgenic plants. ddH_2_O, no template control; ‐, non‐transgenic negative control. (c) qRT‐PCR analysis of the *ZmDA1* and *Zmda1* gene expression levels of the *Zmda1* transgenic lines. (d) qRT‐PCR analysis of the *ZmDAR1* and *Zmdar1* gene expression level of the *Zmdar1* transgenic lines. (e) qRT‐PCR analysis of the *ZmDA1* gene expression level of the *ZmDA1* transgenic lines. (f) qRT‐PCR analysis of the *ZmDAR1* gene expression level of the *ZmDAR1* transgenic lines. Values are the means ± SD;* n* = 6, *P* < 0.05 using Duncan's test. (g) Southern blot analysis of the transgenic plants and wild‐type (WT), M, λ‐*Eco*T14 I digest DNA marker (Takara, Dalian, China). (h) T‐DNA region structure of the plasmid pB7WG2.0‐ Pubi::*Zmda1*‐*bar* and pB7WG2.0‐ Pubi::*Zmdar1*‐*bar*. PUbi, the maize ubiquitin promoter; *Bt*, the *cryIAc* gene from *Bacillus thuringiensis Berline*; P35s, the CaMV35S promoter; *bar*, the phosphinothricin acetyl transferase gene.

The maize B73 cDNA was used as the template to clone the *ZmDA1* and *ZmDAR1* genes. A single‐base mutant was introduced into the gene using the bridge PCR method to produce *Zmda1* and *Zmdar1*. The maize ubiquitin promoter was used to drive the genes, and the herbicide resistance gene *bar* (Thompson *et al*., 1987) was used as select genes for transgenic screening. *ZmDA1*,* ZmDAR1*,* Zmda1* and *Zmdar1* were separately introduced in the DH4866 maize inbred line using *Agrobacterium*‐induced maize shoot‐tip transformation. After herbicide screening and molecular determination, fifteen *Zmda1*, seventeen *Zmdar1*, seven *ZmDA1* and twelve *ZmDAR1* transgenic events were obtained (Figure 2a, b). To examine the expression level of the transgenes in those plants, the total RNA was extracted from the leaves and kernels at DAP15 from various transgenic lines and the WT. qRT‐PCR was used to select high expression lines. As shown in Figure 2, the total transcripts of endogenous *ZmDA1* and transgenic *Zmda1* were significantly increased in the transgenic plants, as well as in the *Zmdar1* transgenic plants (*P* < 0.05, Figure 2c, d). The *ZmDA1* or *ZmDAR1* transcript levels were also significantly increased in the *ZmDA1* or *ZmDAR1* transgenic plants (*P* < 0.05, Figure 2e, f). The PCR‐positive independent transgenic lines were confirmed using Southern blotting. As shown in Figure 2g, signals of exogenous *bar* gene were detected with specific hybridization pattern (the *bar*‐specific probe site is shown in Figure 2h). These result suggested the T‐DNA region had been stably integrated into the genomes of transgenic plants. At last, three independent over‐expression transgenic lines of each construct were selected for the further studies.

### The phenotype of *Zmda1 and Zmdar1* transgenic plants changed slightly during the vegetative growth stage

**Figure 3:**
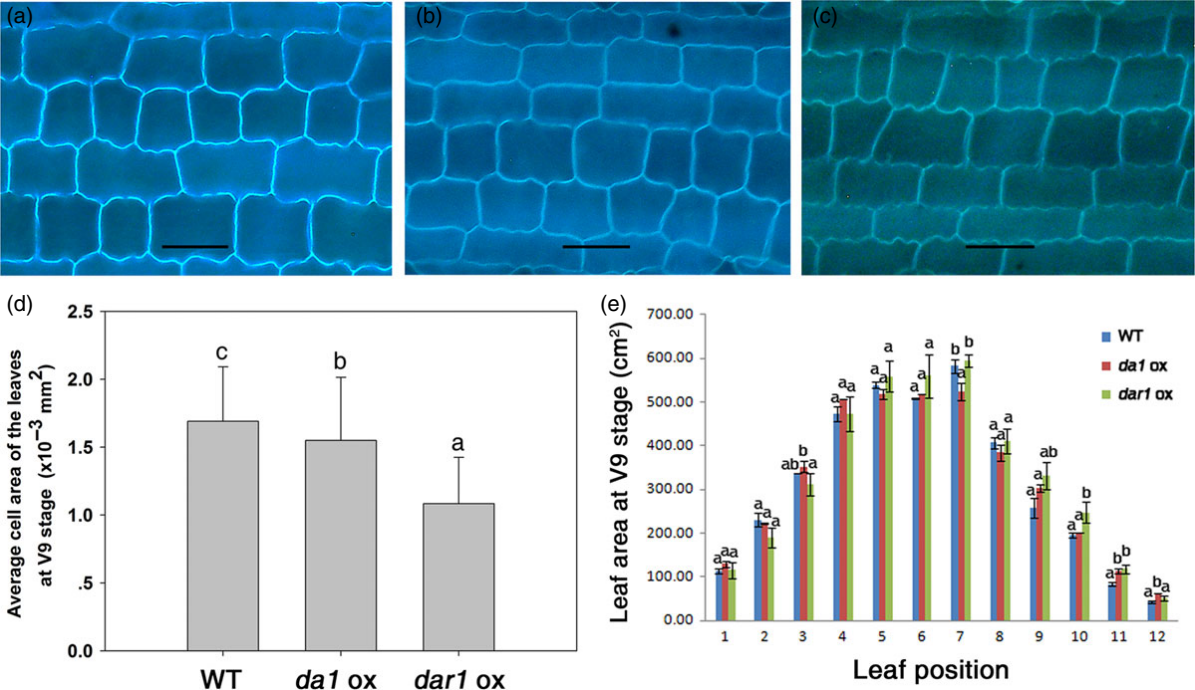
Cellular morphology and leaf area of the transgenic and wild‐type (WT) plants. (a–c) Epidermis cells from a leaf at the V9 stage, scale bar = 50 μm; (a) WT; (b) *Zmda1* transgenic plants; (c) *Zmdar1* transgenic plants; (d) average cell area of the transgenic and the WT plants, n≥200; (e) leaf area from the V9 stage plants. Values are the means ± SD,* n* = 5, *P* < 0.05 using Duncan's test.

Three independent transgenic lines with a single‐copy transgene for each transformation construct at the T3 generation were planted in the field for phenotypic evaluation. The data of plant height, leaf length and leaf width were collected at the V9 stage. There was no obvious difference in plant height, leaf shape or leaf sheath colour between the transgenic and WT plants (Figure S1). As shown in Figure 3e, the area of mature leaves from the *Zmda1* or *Zmdar1* transgenic plants was similar to that of the WT, while the area of immature leaves of the transgenic plants was larger than that of the WT; for example, the tenth leaf of the V9 stage plants had an average leaf area of 201.95 cm^2^ in the *Zmda1* transgenic lines and 248 cm^2^ in the *Zmdar1* transgenic lines compared with an average of 195.4 cm^2^ in the WT. The length of the epidermis cells in the mature leaves from plants at the V9 stage was slightly decreased in the *Zmda1* but increased *Zmdar1* in transgenic lines, and the cell width was remarkably decreased in the *Zmdar1* and *Zmda1* transgenic leaves (Figure 3a, b, c, Figure S2). Compared with that in the WT, the average cell area in transgenic plants was reduced, especially in the *Zmdar1* transgenic lines (*P* < 0.05, Figure 3d). No significant change was observed for the entire leaf area with a decreased cell area; these suggested that the total cell number in the transgenic leaves was greater than that in the WT leaves. Therefore, transgenic *Zmda1* and *Zmdar1* promoted cell division in the leaves but did not significantly affect the leaf phenotype in maize.

### Expressing the *Zmda1* or *Zmdar1* gene improved grain yield in maize by increasing the kernel number and kernel weight

The transgenic plants from the T3 to T5 generation and the WT plants were planted in the field from 2013 to 2015 to determine whether the expression of *Zmda1* or *Zmdar1* would affect the grain yield in maize. In these test, compared with the WT plants, the transgenic plants produced larger ears with an obvious increase in grain weight. When comparing the data from the yield trials for 3 years, as shown in Table 1, both of the transgenic plants with *Zmda1* or *Zmdar1* showed a significant increase in ear length. The increased ear length had more seeds in each ear row and heavier kernels. The grain yield increased as a result of the improvement in the kernel number and kernel weight (Table 1 and Table S2). In the 3 years, the yields were 15%, 16% and 22% higher for the *Zmda1* transgenic lines and 18%, 22% and 21% higher for the *Zmdar1* transgenic lines when compared to the wild‐type. However, the lines that over‐expressed *ZmDA1* or *ZmDAR1* had reduced kernel weight and yields (Table 1 and Table S2). These results indicate that the over‐expression of *Zmda1* or *Zmdar1* improved maize yield, while the over‐expression of *ZmDA1* or *ZmDAR1* decreased maize yield.

**Table 1:** Agronomic trait of WT and transgenic maize in the field in 2014

	Line	Ear length (cm)	Grain number per row	Grain number per ear	100‐grain number (g)	Grain weight per ear(g)	Grain weight per plot (Kg)	Row number per ear
	WT	14.32 ± 0.11 d	31.40 ± 1.37 c	431.31 ± 14.91 e	22.24 ± 0.30 e	91.32 ± 3.99 d	4.38 ± 0.19 d	14.40 ± 0.73 ab
*da1* ox	La19	15.30 ± 0.35 e	34.60 ± 1.69 e	470.92 ± 15.94 f	22.96 ± 0.40 f	98.80 ± 2.90 e	4.74 ± 0.14 e	14.80 ± 0.89 c
	La29	15.46 ± 0.63 e	32.20 ± 1.86 cd	426.06 ± 9.47 e	26.26 ± 0.30 j	105.40 ± 3.44 fg	5.06 ± 0.16 fg	14.00 ± 0.00 ab
	La30	15.80 ± 0.98 e	34.80 ± 2.11 e	476.72 ± 18.58 f	23.30 ± 0.22 fg	101.40 ± 2.98 efg	4.87 ± 0.14 efg	14.40 ± 1.37 ab
*DA1* ox	LA12	11.39 ± 0.24 ab	25.00 ± 0.58 a	342.94 ± 4.80 ab	18.92 ± 0.41 cd	65.53 ± 0.99 b	3.15 ± 0.05 b	14.00 ± 0.00 ab
	LA17	11.62 ± 0.28 b	25.83 ± 1.07 ab	350.69 ± 7.80 abc	18.22 ± 0.27 b	65.28 ± 2.40 b	3.13 ± 0.12 b	13.60 ± 0.73 a
	LA23	11.05 ± 0.21 a	24.50 ± 1.26 a	331.75 ± 11.32 a	17.23 ± 0.33 a	59.35 ± 1.50 a	2.85 ± 0.07 a	13.60 ± 0.73 a
*dar1* ox	Lr38	15.52 ± 0.18 e	34.00 ± 2.58 de	455.01 ± 28.33 f	23.62 ± 0.25 g	99.40 ± 7.25 ef	4.77 ± 0.35 ef	14.40 ± 0.73 ab
	Lr44	15.82 ± 0.40 e	35.20 ± 2.11 e	470.21 ± 28.78 f	24.94 ± 0.48 i	106.40 ± 7.59 g	5.11 ± 0.37 g	14.60 ± 0.73 ab
	Lr62	14.58 ± 0.13 d	37.80 ± 0.89 f	476.32 ± 27.52 f	24.42 ± 0.22 h	99.62 ± 7.44 ef	4.78 ± 0.36 ef	14.40 ± 0.73 ab
*DAR1* ox	LR1	12.52 ± 0.18 c	27.67 ± 1.25 b	374.02 ± 15.52 d	19.23 ± 0.23 d	71.94 ± 3.15 c	3.45 ± 0.15 c	13.60 ± 0.73 a
	LR4	11.92 ± 0.21 b	26.33 ± 1.80 ab	365.90 ± 18.18 bc	18.95 ± 0.31 d	69.36 ± 4.09 bc	3.33 ± 0.20 bc	14.20 ± 0.37 ab
	LR11	11.72 ± 0.18 b	26.00 ± 1.73 ab	359.00 ± 13.09 bc	18.52 ± 0.38 bc	68.06 ± 3.28 bc	3.27 ± 0.16 bc	14.00 ± 0.00 ab

Values are mean ± SD and labelled with letter are significantly different at *P* < 0.05 by Duncan's test. *n* = 18.

**Figure 4:**
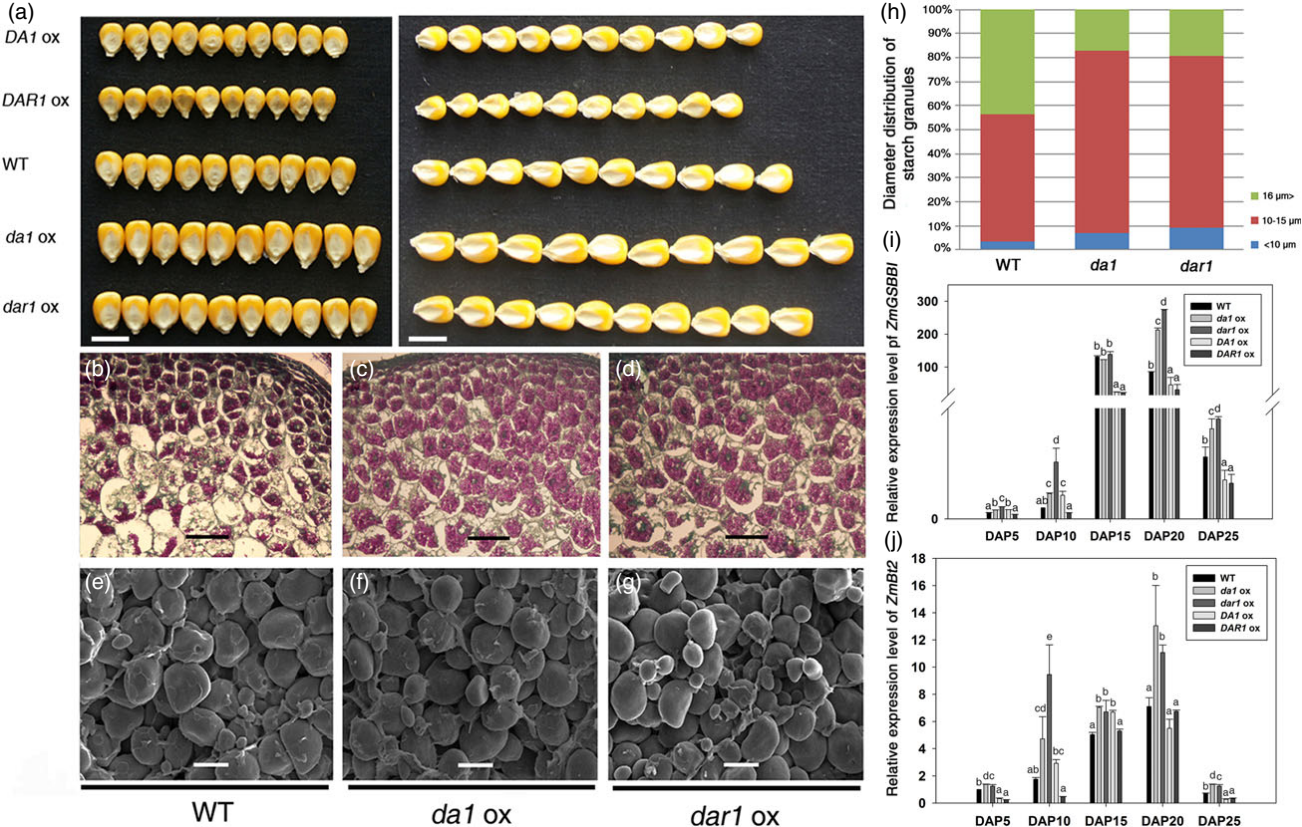
Over‐expression of *Zmda1* and *Zmdar1* increased seed size, modified starch granule morphology and starch synthesis, and enhanced the expression levels of *ZmGBSSI* and *ZmBt2*. (a) Picture of the kernels from transgenic and wild‐type (WT) lines. Scale bar = 1 cm. (b, c, d) Paraffin section and Schiff reaction staining of the endosperm cells, scale bar = 100 μm. (e, f, g) Scanning electronic micrographs of farinaceous albumen starch granules, scale bar = 10 μm. (b, e) WT control; (c, f) *Zmda1* transgenic lines; (d, g) *Zmdar1* transgenic lines. (h) Comparison of the starch granule diameter, n≥200. (i) *ZmGBSSI* expression levels in the transgenic and the WT plants; (j) *ZmBt2* expression levels in the transgenic and the WT plants. Values are the means ± SD;* n* = 6, *P* < 0.05 using Duncan's test.

The grain yield depends on the endosperm weight in cereal crops. Increased kernel weights result from the higher endosperm and/or embryo weight in maize. The seed sizes were increased in the *Zmda1* and *Zmdar1* transgenic plants (Figure 4a). The mass ratio of the endosperm to embryo increased about 10% in the *Zmda1* and *Zmdar1* transgenic lines when compared with that of the WT, and the embryo weight did not show large variation (Table 2). Therefore, the greater kernel weight primarily resulted from the increased endosperm weight in these plants.

**Table 2:** Kernel traits of transgenic maize and wild‐type

	Starch content (%)	Endosperm weight (mg)	Embryo weight (mg)	Endosperm/embryo	Endosperm area (mm^2^)	Endosperm cell number	Endosperm cell area (μm^2^)
WT	61.50 ± 1.90 a	201.37 ± 2.41 a	51.52 ± 0.86 b	3.91 ± 0.08 a	18.93 ± 0.26 a	4632.07 ± 128.29 a	4088.51 ± 65.24 a
La19	67.41 ± 3.37 c	228.71 ± 2.26 c	53.04 ± 0.51 c	4.31 ± 0.08 c	19.62 ± 0.13 b	4851.35 ± 245.27 b	4055.64 ± 204.56 a
La29	67.20 ± 1.73 c	261.56 ± 2.49 f	57.78 ± 0.65 e	4.53 ± 0.09 d	21.15 ± 0.50 d	5324.30 ± 137.94 d	3976.85 ± 161.89 a
La30	70.50 ± 0.59 d	248.20 ± 0.70 e	57.73 ± 1.13 e	4.30 ± 0.09 c	19.34 ± 0.07 b	4948.71 ± 92.56 bc	3908.63 ± 65.75 a
Lr38	69.42 ± 1.27 cd	242.02 ± 1.26 d	56.31 ± 1.09 d	4.30 ± 0.06 c	19.45 ± 0.41 b	5026.80 ± 178.33 bcd	3871.78 ± 77.96 a
Lr44	71.07 ± 1.10 d	218.51 ± 3.39 b	49.37 ± 1.26 a	4.43 ± 0.08 d	20.13 ± 0.31 c	5163.07 ± 223.36 cd	3906.97 ± 206.94 a
Lr62	64.52 ± 0.86 b	225.80 ± 0.89 c	54.32 ± 0.33 c	4.16 ± 0.03 b	19.98 ± 0.20 c	5018.66 ± 160.15 bcd	3986.33 ± 144.59 a

Values are mean ± SD and labelled with letter are significantly different at *P* < 0.05 by Duncan's test. *n* = 9.

### *Zmda1* and *Zmdar1* transgenic plants had increased starch content and smaller starch granules in kernels

Starch is the major component of maize endosperm and substantially affects crop yield. We examined the total starch content of transgenic T3, T4 and the WT kernels. The total starch content was significantly increased by approximately 11% in the *Zmda1* transgenic lines and 14% in the *Zmdar1* transgenic lines (Table 2). The staining of the endosperm cell at 12DAP with Schiff's reagent showed an increased starch content in the endosperm of the transgenic plants during the grain‐filling stage. This demonstrates that the starch accumulation was enhanced in the *Zmda1* or *Zmdar1* transgenic plants (Figure 4b, c, d).

Scanning electron microscopy analyses showed that the starch granules were more rounded with an uneven size in the *Zmda1* or *Zmdar1* transgenic kernels (Figure 4e, f, g). The starch granules were classified into three groups according to the diameter by Franco (Franco *et al*., 1992): (i) larger than 16 μm; (ii) between 15 and 10 μm; and (iii) smaller than 10 μm. The statistics data for the transgenic kernels showed that the starch granules of group (i) substantially decreased and the average diameter was also reduced. However, in group (ii) and (iii), the population of the starch granules significantly increased in the transgenic lines compared with the WT (Figure 4h). Group (i) has a smaller proportion of starch granules; thus, the average diameter of the starch granules decreased in the *Zmda1* and *Zmdar1* transgenic lines.

In the developing maize kernels, sucrose is converted to glucose‐1‐phosphate; glucose‐1‐phosphate then reacts with ATP via ADP‐glucose pyrophosphorylase (the subunits of which are encoded by the *ZmSh2* (*ZmShrunken2*) and *ZmBt2* (*ZmBrittle2*) genes) to yield ADP‐glucose, which is the key substrate for starch synthesis. Through the action of starch synthase and branching enzymes, the glucose from this high‐energy intermediate is polymerized. The starch synthase enzymes can be divided into two categories: soluble enzymes and enzymes bound tightly to the starch grain. The *ZmGBSSI* (*ZmWx1*) gene encoded starch granule‐bound ADP‐glucose glucosyltransferase (starch synthase I), which is responsible for amylose synthesis and influences the starch content in the endosperm. *ZmGBSSI* and *ZmBt2* played a very important role in starch synthesis and showed a significant difference between the transgenic and WT. The relative expression levels of the genes were measured using qRT‐PCR. Both genes were up‐regulated in the *Zmda1* and *Zmdar1* transgenic lines and down‐regulated in the *ZmDA1* and *ZmDAR1* over‐expression lines (Figure 4i, j). These results suggest that the expression of the *Zmda1* or *Zmdar1* gene in maize enhances starch synthesis.

### *Zmda1* and *Zmdar1* transgenic ovules have a more developed basal endosperm transfer cell layer (BETL) and greater soluble sugar content

**Figure 5:**
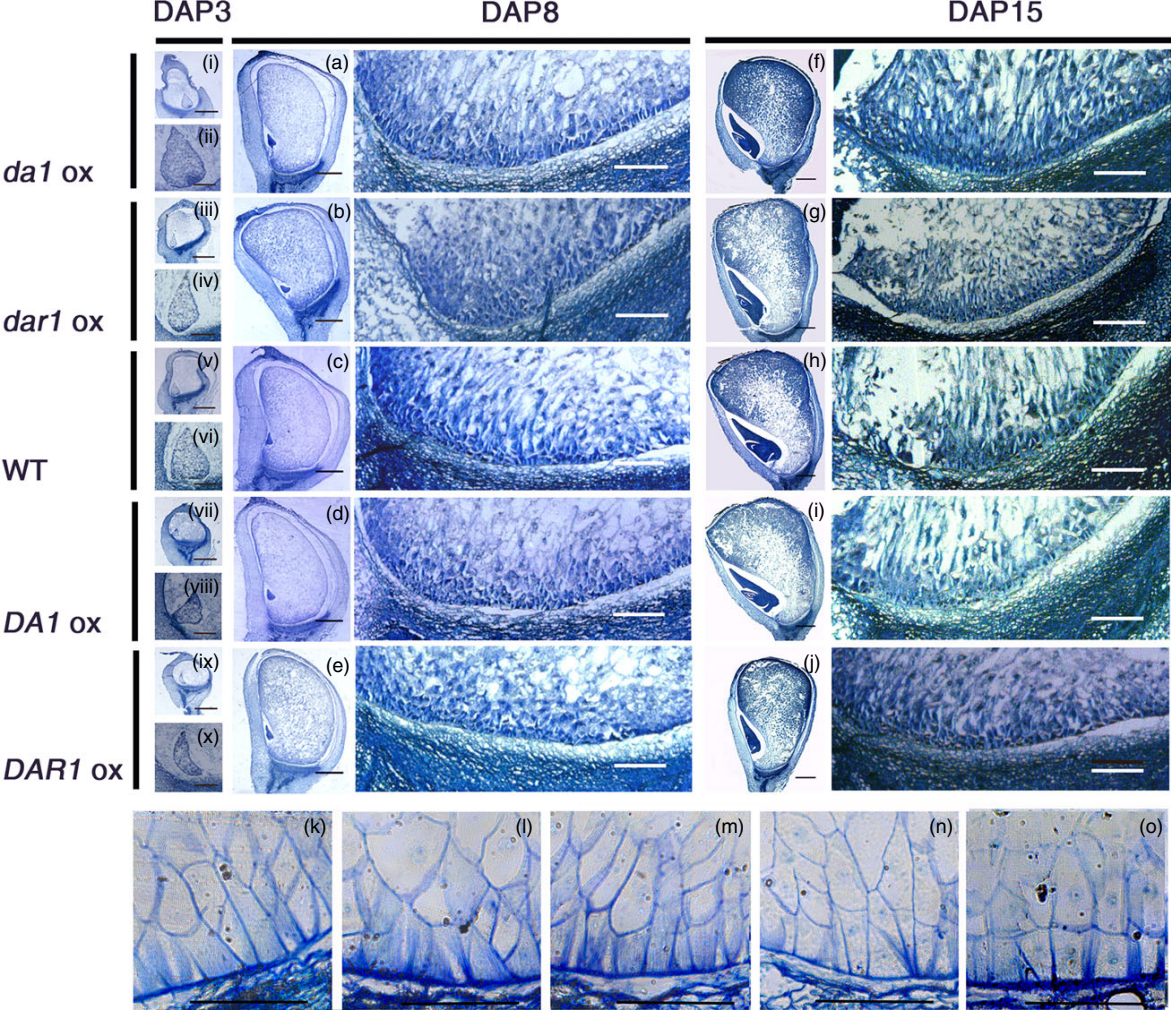
The paraffin section and semi‐thin section of maize kernels at different stages in the transgenic and wild‐type (WT) plants. (i, iii, v, vii, ix) Paraffin sections of the whole kernel at DAP3 from different transgenic and the WT events, scale bar = 1 mm; (ii, iv, vi, viii, x) larger image of the free nuclear endosperm at DAP3, scale bar = 250 μm; (a–e) the left part was the paraffin section of the whole kernel at DAP8, scale bar = 1 mm; the right part was larger image of the basal endosperm transfer cell layers (BETLs) of the DAP8 maize kernels, scale bar = 250 μm; (f–j) the left part was the paraffin section of the whole kernel at DAP15, scale bar = 1 mm; the right part was larger image of the basal endosperm transfer cell layers (BETLs) of the DAP15 maize kernel, scale bar = 250 μm. (k–o) the semi‐thin section of maize kernels at DAP12, (k) WT; (l) *Zmda1* transgenic line; (m) *Zmdar1* transgenic line; (n) *ZmDA1* transgenic line; (o) *ZmDAR1* transgenic line, scale bar = 50 μm.

**Figure 6:**
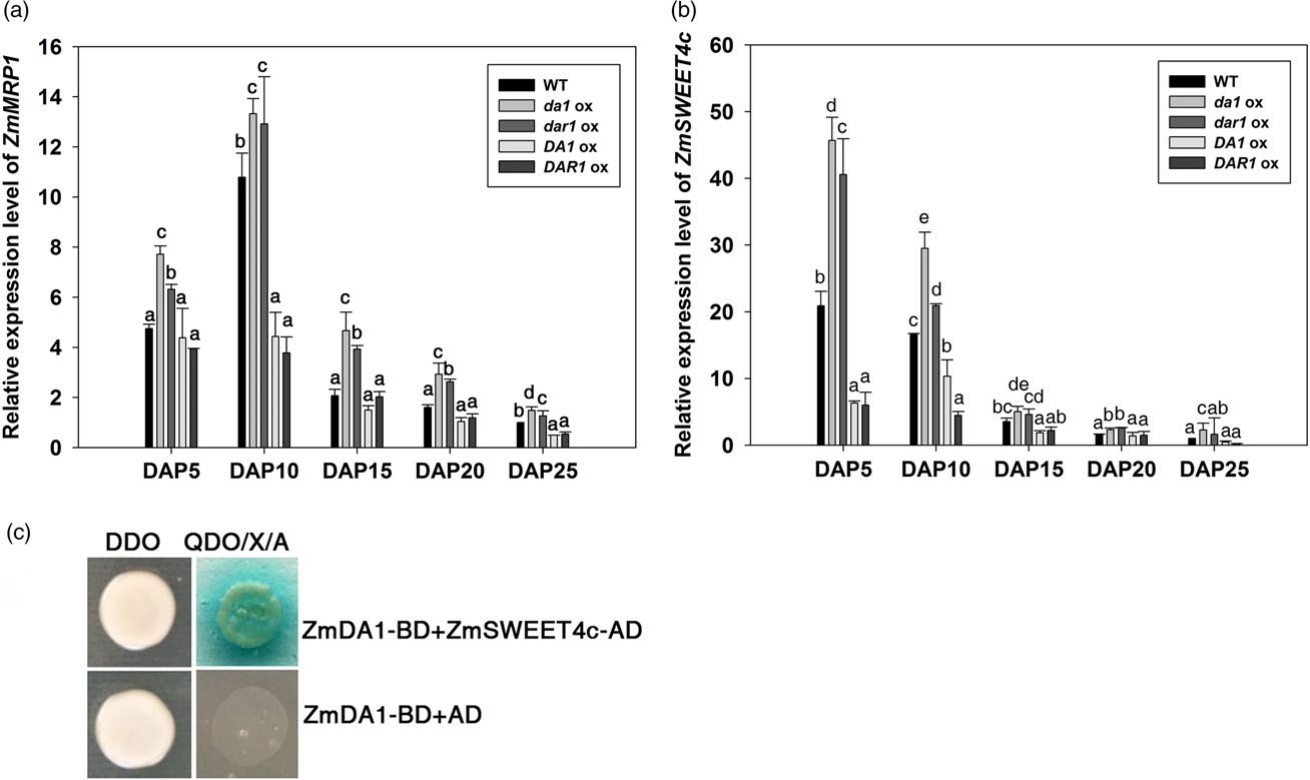
Over‐expression of *Zmda1* or *Zmdar1* regulated the expression levels of *ZmMRP1 and ZmSWEET4c*. (a) *ZmMRP1* expression levels in the transgenic and the WT plants; (b) *ZmSWEET4c* expression levels in the transgenic and the WT plants. Values are the means ± SD;* n* = 6, *P* < 0.05 using Duncan's test. (c)The Y2H analysis of ZmDA1 and ZmSWEET4c, DDO, the ‐Leu‐Trp dropout supplements, QDO/X/A, ‐Ade‐His‐Leu–Trp dropout supplements containing X‐α‐gal and 3‐AT.

The anatomical morphology of the kernels was observed to ascertain the mechanism of the increased grain yield and greater starch content. During the early stage of endosperm development, the primary endosperm nucleus undergoes several rounds of division without cytokinesis to generate a large number of free nuclei organized at the periphery of the central cell. At DAP3, in the *Zmda1* and *Zmdar1* transgenic plant cells, the base of free nuclear endosperm showed higher‐density protoplasm than the *ZmDA1* and *ZmDAR1* over‐expression plants and the WT (Figure 5i to x). Faster embryo development at early stage was observed in the *Zmda1* transgenic lines. Microscopic image showed that the transfer cells were well developed in the *Zmda1* or *Zmdar1* transgenic seed when compared with the wild‐type (Figure 5k, l, m). But in the *ZmDA1* or *ZmDAR1* over‐expression seed, the transfer cells showed a poor development (Figure 5k, n, o). We speculated that endosperm development was faster in the *Zmda1* and *Zmdar1* transgenic lines than in the WT which may be due to the earlier formation of transfer cells and the well‐developed cell wall ingrowths, and combined with changes in the number of endosperm cells. The BETL cells of the *Zmdar1* transgenic lines were smaller than those of the WT, and there was also an increase in the number of cells (Figure 5m, Table 2). This specialized region functions in metabolite transport and provides an indirect connection with the maternal tissues. Both traits in transgenic plants suggest a larger cell wall area to transport more nutrients into the inner zone of the endosperm. The transcriptional regulator *ZmMRP1* (MYB related protein 1) plays an important role in transfer cell development. We detected the relative expression level of *ZmMRP1* in various transgenic lines and the WT. As shown in Figure 6a, the *ZmMRP1* in *Zmda1* or *Zmdar1* transgenic plants was up‐regulated relative to that in the WT. Thus, the expression of *Zmda1* or *Zmdar1* enhanced the expression of *ZmMRP1* and transfer cell development. When we measured the soluble sugar content in the developing kernels using anthrone‐sulphuric acid colorimetric method, the soluble sugar levels were different between transgenic plants and the WT. Compared with the WT, the sugar content was higher in the *Zmda1* or *Zmdar1* transgenic plants and lower in the *ZmDA1* or *ZmDAR1* over‐expression line at DAP5 and DAP10 (Table 3). However, the statistic difference was not significant between *Zmdar1* transgenic lines and the wild‐type at DAP5. *ZmSWEET4c*, which belongs to the SWEET sugar transporter gene family, has been reported to mediate glucose import and induce the expression of *ZmMRP1* (Sosso *et al*., 2015). In the *Zmda1* and *Zmdar1* transgenic plants, *ZmSWEET4c* was up‐regulated during the early stage of seed development (Figure 6b); from these, one could infer that the *ZmDA1* and *ZmDAR1* genes played a role in the regulation of sugar transport and transfer cell development.

**Table 3:** The soluble sugar content in transgenic maize and wild‐type

	DAP5	DAP10	DAP15	DAP20	DAP25
WT	187.77 ± 1.52 c	128.98 ± 1.12 b	20.63 ± 0.91 a	12.02 ± 1.56 a	15.90 ± 0.03 c
*da1* ox	199.72 ± 6.55 d	144.42 ± 1.71 c	31.16 ± 0.35 b	35.46 ± 0.06 bc	12.94 ± 0.76 b
*dar1* ox	189.43 ± 7.25 c	152.81 ± 3.34 d	30.02 ± 1.55 b	36.56 ± 2.71 bc	8.82 ± 1.46 a
*DA1* ox	163.29 ± 4.36 b	114.35 ± 3.35 a	44.50 ± 2.29 d	30.72 ± 8.09 b	25.48 ± 1.04 e
*DAR1* ox	150.54 ± 1.25 a	111.20 ± 6.83 a	35.37 ± 0.05 c	39.11 ± 7.14 c	20.15 ± 0.07 d

The unit of the data is mg/g DW. Values are mean ± SD and labelled with letter are significantly different at *P* < 0.05 by Duncan's test. *n* = 3.

## Discussion

The ubiquitin‐proteasome pathway plays a crucial role in protein metabolism and is implicated in the regulation of many biological processes, such as cell cycle control, DNA damage response. The pathway has recently been shown to regulate plant organ and seed sizes (Li and Li, 2014). In *Arabidopsis*, the ubiquitin receptors *AtDA1* and *AtDAR1* act redundantly to control seed size by restricting cell proliferation in maternal integuments (Li *et al*., 2008). Studies in wild soybean suggest that the DA1 and DA1‐related gene family members may have different functions in different species (Zhao *et al*., 2014). In this study, *ZmDA1* and *ZmDAR1* were identified as the homologous genes of *AtDA1* and *AtDAR1*. *ZmDA1* and *ZmDAR1* transgenic plants showed the decreased kernel weight and slowed growth, while the transgenic plants with mutated *Zmda1* or *Zmdar1* genes showed the increased kernel number and kernel size. The expression of the mutated genes was driven by the ubiquitin promoter in the transgenic plants. Notably, the total cell number in the leaves of *Zmda1* and *Zmdar1* transgenic plants was increased; however, the mature leaf area did not show obvious variations, and the phenotype of the transgenic plants was similar to that of the WT plants except in the case of the ear. *ZmDA1* and *ZmDAR1* have been suggested to play a role in determining the final seed size because the over‐expressed lines produced smaller kernels through their influence on sugar transport into the endosperm. Additionally, there were no effects on organ size in maize (Figures S1, S3‐S5), which indicates that there were delicate differences between ZmDA1/ZmDAR1 and AtDA1/AtDAR1. We speculate that the substrates degraded in the DA1 and DAR1 regulating pathway may differ between the two species despite similar gene sequences in *Arabidopsis* and maize.

This study found that *ZmDA1* and *ZmDAR1* have a close evolutionary relationship; however, there are subtle differences. The expression pattern analysis showed that *ZmDAR1* has a greater transcriptional level in the kernels after pollination than *ZmDA1* (Figure 1b, c). The *ZmDAR1* gene had a high expression level in the endosperm and a lower expression in the embryo; however, this result was opposite in the expression patterns of *ZmDA1*. This suggests that *ZmDAR1* may primarily function in maize endosperm development and that *ZmDA1* may play an important role in embryo development. In the 3‐year field test, we found that the *Zmdar1* transgenic lines showed a better yield phenotype (Table 1), including more kernels per ear and greater starch content, than *Zmda1* transgenic and the WT plants (Table 2). These results indicate that *ZmDAR1* plays an important role in regulating endosperm development. A further study of the *Zmda1* and *Zmdar1* mutants will verify this speculation.

In the study performed by Wang as well as in the current work, the enhanced grain yield was due to both the increased seed number and the increased grain weight (Wang *et al*., 2012). Why did the *Zmda1* and *Zmdar1* transgenic plants have better yields than the WT plants? In *Arabidopsis*, the DA1R358K mutant dramatically increases seed and organ size, and the mutant protein has a negative effect on the function of DA1 and DAR1 proteins (Li *et al*., 2008). As shown in the evolutionary tree (Figure 1a), ZmDA1 and ZmDAR1 have no fewer than one paralog in maize. In the *Zmda1* and *Zmdar1* transgenic plants, perhaps the mutated proteins must compete with partners to form an impaired complex, which restrains the function of DA1 or DAR1 proteins, and led to the accumulation of substrates that should have been degraded. These substrates may play important roles in cell proliferation, sugar transport or starch synthesis pathways. In recently study, the AtDA1 and AtDAR1 were identified as peptidase which could be activated by multiple ubiquitylation (Dong *et al*., 2017). The activated AtDA1 cleaves several growth regulators, such as AtTCP15, AtTCP22 and AtUBP15, and then affects the cell proliferation in *Arabidopsis*. The cleavage activity of the R358K mutant protein was decreased to 30% when compared with that of wild‐type DA1. Using a yeast two‐hybrid system, ZmSWEET4c was identified when ZmDA1 was used as bait (Figure 6c). It suggested ZmSWEET4c might be a potential substrate of ZmDA1. The ZmSWEET4c was accumulated because of the decreased peptidase cleavage activity in the *Zmda1* transgenic plants. In the early stage of seed development, background level glucose induced the expression of *ZmINCW2* and *ZmSWEET4c*. ZmINCW2 cleaves sucrose, which was unloaded from the phloem termini. During kernel development, most of the sucrose from the maternal tissue at the base of the endosperm is cleaved to glucose and fructose by invertase. Then, ZmSWEET4c transports the hexaose into the developing seed. In the *Zmda1* transgenic ovules, the accumulation of ZmSWEET4c might increase the hexaose content in endosperm and improve the ratio of hexaose to sucrose which is helpful to the cell proliferation at the early stage of the seed development (Weber *et al*., 1997). And the increased glucose concentration could activate the expression of *ZmMRP1*, which is a core transcription regulator in basal endosperm transfer cell formation (Sosso *et al*., 2015). Sufficient development of the transfer cells in the kernels would lead to the increased nutrient transport into the endosperm; thus, more kernels would have the opportunity to develop, especially those located at the top of the ears, which are aborted under normal conditions.

Interestingly, transgenic maize over‐expressing *ZmINCW2* showed a similar phenotype with the *Zmda1* and *Zmdar1* over‐expression lines, which had an increased seed weight and starch content. Notably, both the *ZmINCW2* transgenic plants and the *Zmda1* and *Zmdar1* transgenic plants had a reduced average diameter of the starch granule (Li *et al*., 2013). The smaller starch granule provided a larger superficial area that could bind more proteins for starch synthesis. The increased sugar content in *Zmda1* or *Zmdar1* transgenic kernels could be speculated to enhance the expression of important enzyme genes in the starch synthesis pathway. The weight of the endosperm in the *Zmda1* and *Zmdar1* transgenic plants was increased to 13.7%–30.2% compared with that of the WT (Table 2). The starch content was also higher in the *Zmda1* and *Zmdar1* over‐expression lines when compared with the WT. In the *Zmda1* and *Zmdar1* over‐expression lines, developing kernels showed increased soluble sugar content, and the expression levels of *GBSSI* and *Bt2* were up‐regulated. The increased accumulation of the two genes transcripts would enhance starch synthesis (Hanashiro *et al*., 2008; Li *et al*., 2011). Previous studies showed that sugars, such as sucrose, glucose and fructose, could induce *GBSSI* expression in potato (Visser *et al*., 1991). In the *Zmda1* and *Zmdar1* transgenic lines, the expressions of these genes were up‐regulated, and the result was opposite in the *ZmDA1* or *ZmDAR1* over‐expression lines. However, in the semi‐RT PCR assay, the *Sh2* that encodes the large subunit of ADP‐glucose pyrophosphorylase was not varied with high expression levels in both the transgenic and WT plants. The higher starch content in the *Zmda1* and *Zmdar1* transgenic kernels could due to the increase of the soluble sugar that was imported into the endosperm and the improved starch synthesis. The connection between the transcription of the *GBSSI* and *Bt2* genes and soluble sugar concentrations warrants further exploration.

In conclusion, we successfully obtained maize *Zmda1* and *Zmdar1* transgenic lines with improved grain yield and starch content. The phenotypic change in the maize transgenic plants was related to an improved soluble sugar content and the improved development of the basal endosperm transfer cell layer (BETL) in developing kernels. Moreover, *ZmDA1* or *ZmDAR1* may play a role in the regulation of sugar transport and starch synthesis.

## Experimental procedures

### Bioinformatics analysis of the DA1 gene family

Sequences of the eight members of the DA1 gene family proteins in *Arabidopsis* were obtained from TAIR (http://www.Arabidopsis.org/). The sequences were used as bait to acquire maize DA1 and DARs proteins. BLASTP was performed in the Gramene database (http://www.gramene.org/). Amino acid sequences of the proteins in *Arabidopsis* and maize were aligned using ClustalW (Thompson *et al*., 2002), and the phylogenetic tree was generated using MEGA5 software (Tamura *et al*., 2011). The neighbour‐joining method was used with bootstrap values from 1000 replicates at each branch. The gene expression pattern data of the *Arabidopsis* and maize *DA1* gene family were obtained from Bar (http://bar.utoronto.ca/) (Sekhon *et al*., 2011; Winter *et al*., 2007).The heatmap of the maize *DA1* gene family was constructed using the Heatmapper Plus program (http://bar.utoronto.ca/ntools/cgi-bin/ntools_heatmapper_plus.cgi).

### Gene expression pattern determination and qRT‐PCR

The seeds of the maize elite inbred DH4866 line were grown in the field. The developing organs and kernels were harvested and frozen with liquid nitrogen and stored at −80 °C for RNA extraction. Total RNA was extracted using a TRIzol kit (Takara, Dalian, China). cDNA was synthesized using a reverse transcription kit (Takara, Dalian, China) with 500 ng of total RNA as the template. The specific primer pairs of *ZmDA1* and *ZmDAR1* were used to detect the gene expression pattern. The maize *ACTIN1* gene served as the reference control. The decimal dilution cDNA was used as the template. All of the primer pairs used in this study are listed in Table S3. qRT‐PCR was performed using the ABI7500 (ABI, California, American) and SYRB^®^ *Premix Ex Taq* (Takara, Dalian, China) systems. The fold changes in the target genes were calculated using the 2^−▵▵Ct^ method.

### Clone of *Zmda1* and *Zmdar1* and construction of plant expression vectors

Full‐length fragments of *ZmDA1* and *ZmDAR1* were cloned using PCR and cDNAs from maize inbred line B73 with primer pairs *ZmDA1*F and *ZmDA1*R as well as *ZmDAR1*F and *ZmDAR1*R. *ZmDA1*F and m*ZmDA1*r as well as m*ZmDA1*f and *ZmDA1*R bridge PCR primer pairs were used in the first PCR amplification to introduce a G to A single‐base mutant in two fragments. *ZmDA1*F and *ZmDA1*R were used to join the two fragments together. A similar method was used to introduce a G to A single‐base mutant at the 966 bp site in *ZmDAR1*. The first PCR amplification was performed with the following parameters: 95 °C for 5 min; 35 cycles of 95 °C for 1 min, 56 °C for 1 min, 72 °C for 1 min; and 72 °C for 5 min. The second PCR amplification was similar to the first but with extension duration of 2 min. The fragments of *Zmda1*,* Zmdar1*,* ZmDA1* and *ZmDAR1* were cloned into a donor vector that cascaded with a *bt* gene and a *bar* gene; the fragment was then recombined with the pB7WG2.0 vector using a LR gateway reaction with the LR Clonase enzyme kit (Invitrogen, California).

### Maize transformation and transgenic line production

The *Agrobacterium*‐induced maize shoot‐tip transformation followed a previously described protocol (Li *et al*., 2011). Maize inbred line DH4866 was used as the plant receptor. The T1 transgenic plants were detected using Basta herbicide (0.4% effective concentration). The plants showing Basta resistance were selected to self‐pollinate. T2 and T3 transgenic plants were self‐pollinated to produce progeny.

Genomic DNA was isolated using the CTAB protocol from young T2 transgenic plant leaves. The exogenous *bar* gene was detected using specific primers. PCR amplification was performed with the following parameters: 95 °C for 5 min; 35 cycles at 95 °C for 1 min, 56 °C for 1 min, 72 °C for 1 min; and 72 °C for 5 min. The total RNA was extracted from the kernels at 15DAP in the transgenic plants. The transcriptional level was detected using qRT‐PCR as previously described.

Totally 45 μg genomic DNA of the T3 transgenic maize and wild‐type leaves was digested by *Spe*I at 37 °C over night. The well‐digested DNA was fractionated on 0.8% agarose gels and transferred to nylon membranes (Roche, Mannheim, Germany). The PCR product of *bar* gene fragment was labelled with digoxigenin‐dUTP (Roche). Probe hybridization and detection were using the DIG NBT/BCIP kit for nucleic acids (Roche) following the manufacturer's instructions.

### Field trials

To obtain the yield parameters of the transgenic lines, field trials were carried out in the field in Jinan (Shandong province, China) in 2013 to 2015. T3 and T5 generation plants were grown in three replicates in four‐row plots (48 plants per plot). The plot was 2.5 m in length and 2 m in width with 20 cm between the plants in each row. The measured values were collected from three replicates with six plants per replicate. The mature ears were harvested and dried to a constant weight at 48 °C; the ears were measured for length, the kernel number and rows were counted, and the ears were weighed. The 100‐grain weight was determined in three replicates for each plot. Ten seeds from each ear were sampled to measure the weight of the endosperm and embryo with three replicates of each.

### Histological observation

To determine the cell size and number, the fully expanded 7th leaf of the maize plants at the V9 stage was obtained. The samples were placed in a destaining solution (75% ethanol and 25% acetic acid) overnight at room temperature. The samples were then transferred into a basic solution (7% NaOH in 60% ethanol) for 15 min at room temperature. An ethanol series (40%, 20% and 10%) was used for rehydration for 15 min. The leaves were then placed in 5% ethanol and 25% glycerol for 30 min. Samples were mounted in 50% glycerol and photographed using a microscope (Olympus BX51, Tokyo, Japan). For the cell area count, at least 200 cells were measured in one biological replicate using ImageJ (Perez and Pascau, 2003), and a total of five biological replicates were used in the test.

The anatomical morphology of the kernels was observed using paraffin sections. The kernels were collected from the ears at 3, 8 and 15 days after self‐pollination and were then fixed in 50% FAA (containing 50% ethanol and 4% formaldehyde) for 24 h. The samples were dehydrated in an ethanol gradient series from 50% to 100%. The samples were then cleared using xylene and were embedded in paraffin. The samples were sectioned at 10 μm thickness, stained with toluidine blue‐O and observed with an Olympus BX51 microscope. To observe the starch content during the grain‐filling stage (12 DAP), starchy endosperm cells were counterstained using Schiff's reagent and fast green.

### Semi‐thin section

The kernels of the transgenic and wild‐type plants were obtained at DAP12. The samples were dissected (3 mm in diameter) to keep the bottom of the kernels. The samples were prepared and fixed as previously described (Monjardino *et al*., 2013). The sample was embedded followed the instructions of the SPI‐Pon812™ Kit (Supplies West Chester, USA). After the polymerization, the semi‐thin sections were obtained by ultramicrotome (RMC Prowertome‐XL&CR‐X Tucson, USA). The sections were stained by 1% toluidine blue‐O and then observed and photographed (Leica DFC450 C, Wetzlar, Germany).

### Soluble sugar content measurement

The kernels were harvested from the ear on different days (5, 10, 15, 25 DAP) after self‐pollination and were then dried to a constant weight at 80 °C in a drier. The soluble sugar was measured as previously described (Reyes *et al*., 2006).

### Starch determination and starch granule observation

The starch content in the dried endosperm of mature kernels was determined following the protocol as previously described (Li *et al*., 2011). A formula was used to calculate the starch content: starch content % = G × 0.9/DW × 100% (where G represents the total glucose content of the endosperm and DW is the weight of the dried endosperm).

The starch granules of the dried endosperm of mature kernels from the WT and transgenic plants were observed using scanning electron microscopy (SEM). The protocol was completed as previously described (Zhao *et al*., 2015). The diameters of the starch granules were measured using ImageJ, and 200 starch granules were measured for each sample with five replicates from each sample.

### Y2H assay

The full‐length cDNA sequences of *ZmDA1* and *ZmSWEET4c* were recombination into the pGBKT7 and pGADT7 separately. The yeast transformation followed the instructions of the yeast transformation kit (Clontech Matchmaler™ Mountain View, USA). The positive clones were screened by the ‐Leu–Trp and ‐Ade‐His‐Leu–Trp dropout supplements (Clontech Mountain View, USA), and the positive clones were coloured by X‐α‐Gal (Solarbio Beijing, China).

### Measurements of cell number and cell area of the endosperm

The kernels were harvested from the middle site of the ears at DAP15. Paraffin‐embedded kernels were cut into 10 μm thickness and were stained by Schiffer's reaction. The cell number and the section area of kernels were measured in WT and transgenic kernels by ImageJ (Perez and Pascau, 2003). The average cell area was calculated by the endosperm area divided by the endosperm cell number in the section. Nine seeds of the each transgenic lines and WT were used in this determination.

### Data analysis

Comparisons between the transgenic and WT plants were made using Duncan's multiple‐range test with a one‐way anova in SPSS (version 22.0.0.0). Standard errors are provided for statistical diagrams as appropriate.

## Supporting information


**Figure S1** The phenotype of the wild‐type (WT) and T4 generation plants during the vegetative growth stage.

**Figure S2** The distribution of cell length and width in transgenic and the wild‐type plants.

**Figure S3** The agronomic traits of the wild‐type (WT), *Zmda1* and *Zmdar1* over‐expression plants in the field.

**Figure S4** The agronomic traits of the wild‐type (WT) and *ZmDA1* and *ZmDAR1* over‐expression plants in the field.

**Figure S5** The agronomic traits of the wild‐type and transgenic plants.

**Table S1** The expression pattern of DA1 gene family of maize and *Arabidopsis*.

**Table S2** Agronomic traits of WT and transgenic maize in the field in 2013 and 2015.

**Table S3** The primer pairs used in this study.
