# Supplementary figures and images for: Over‐expression of mutated ZmDA1 or ZmDAR1 gene improves maize kernel yield by enhancing starch synthesis
Source: Plant Biotechnol J. 2017 Jul 25;16(1):234–44. doi: 10.1111/pbi.12763 (PMC5785342; doi:10.1111/pbi.12763)

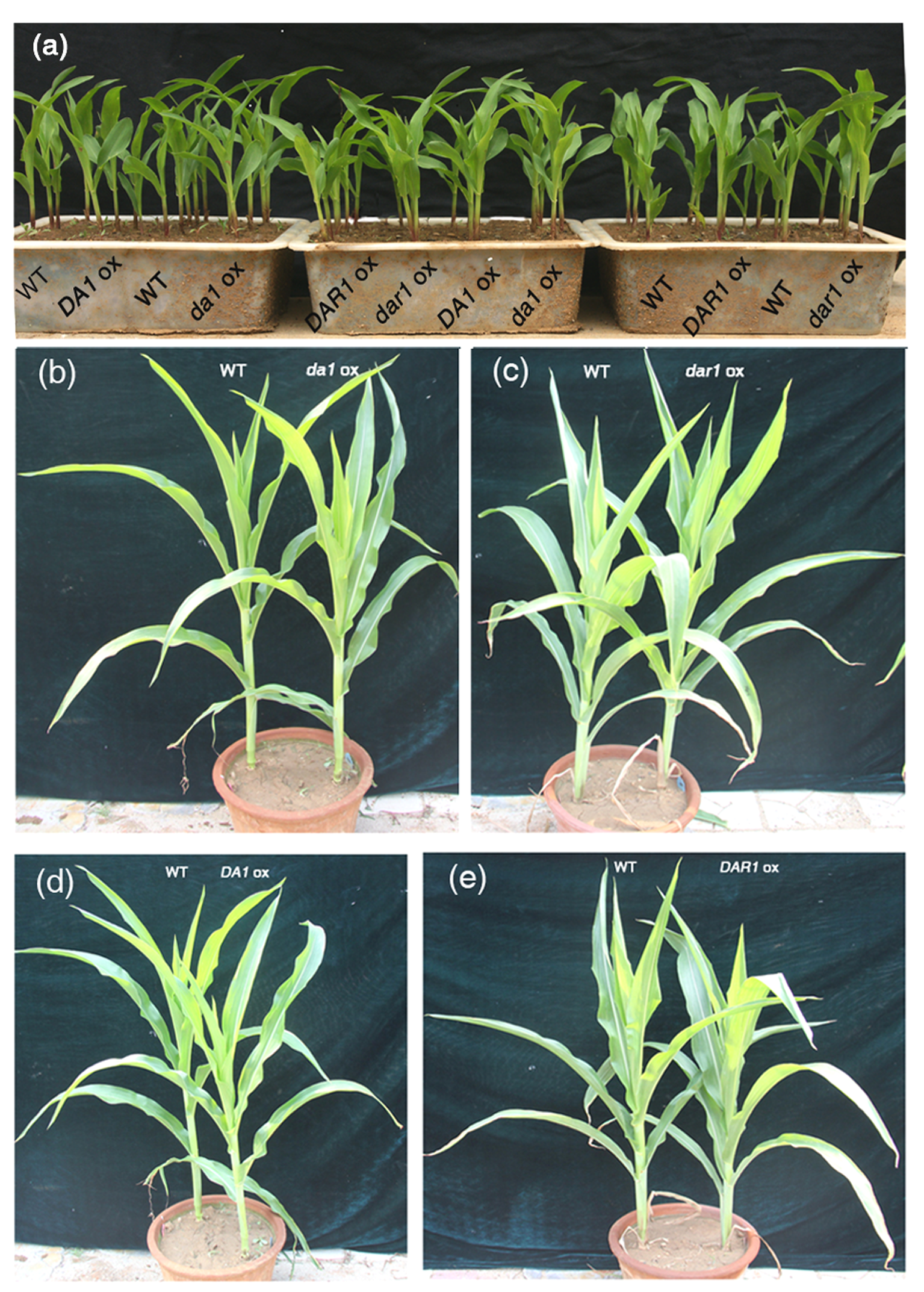

Supplement: Supplementary file 1 — Figure S1 The phenotype of the wild‐type (WT) and T4 generation plants during the vegetative growth stage. [file PBI-16-234-s007.tif]

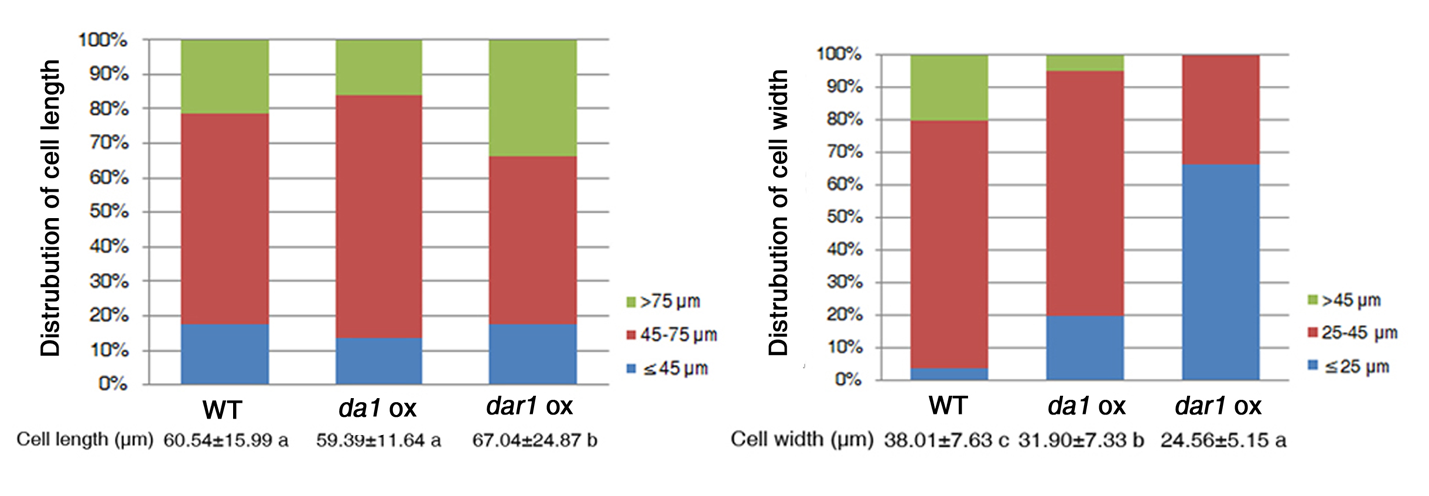

Supplement: Supplementary file 2 — Figure S2 The distribution of cell length and width in transgenic and the wild‐type plants. [file PBI-16-234-s006.tif]

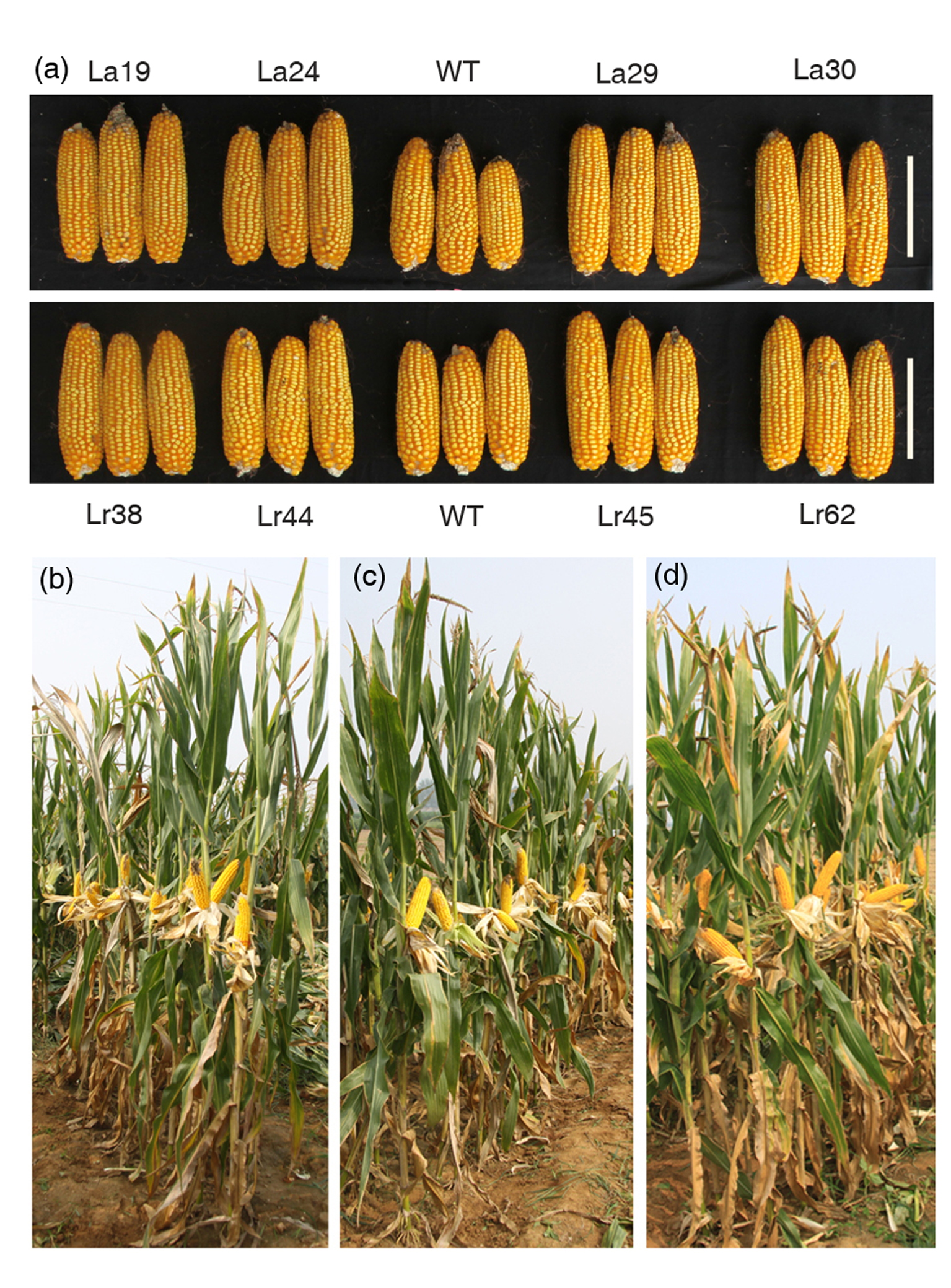

Supplement: Supplementary file 3 — Figure S3 The agronomic traits of the wild‐type (WT), Zmda1 and Zmdar1 over‐expression plants in the field. [file PBI-16-234-s005.tif]

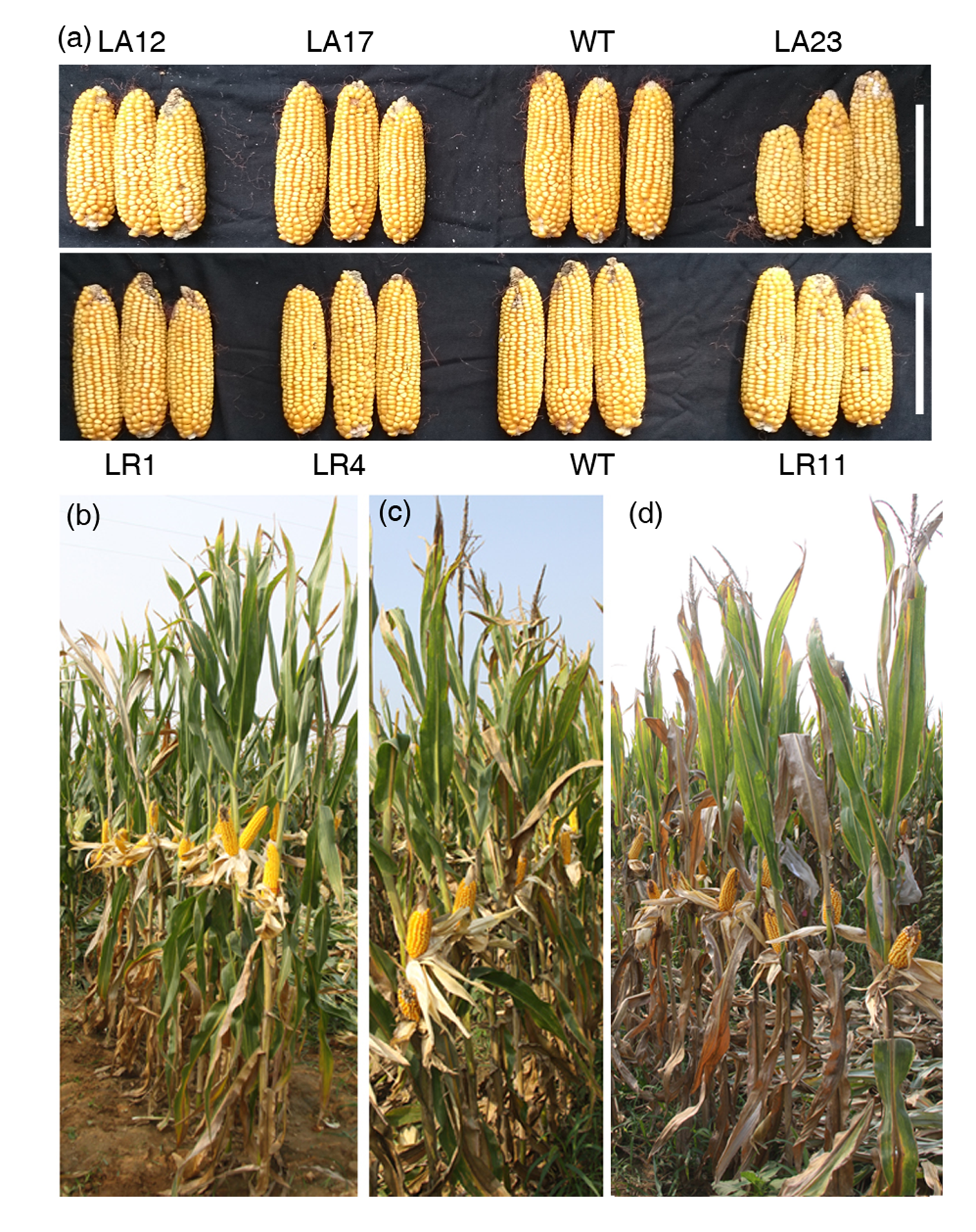

Supplement: Supplementary file 4 — Figure S4 The agronomic traits of the wild‐type (WT) and ZmDA1 and ZmDAR1 over‐expression plants in the field. [file PBI-16-234-s008.tif]

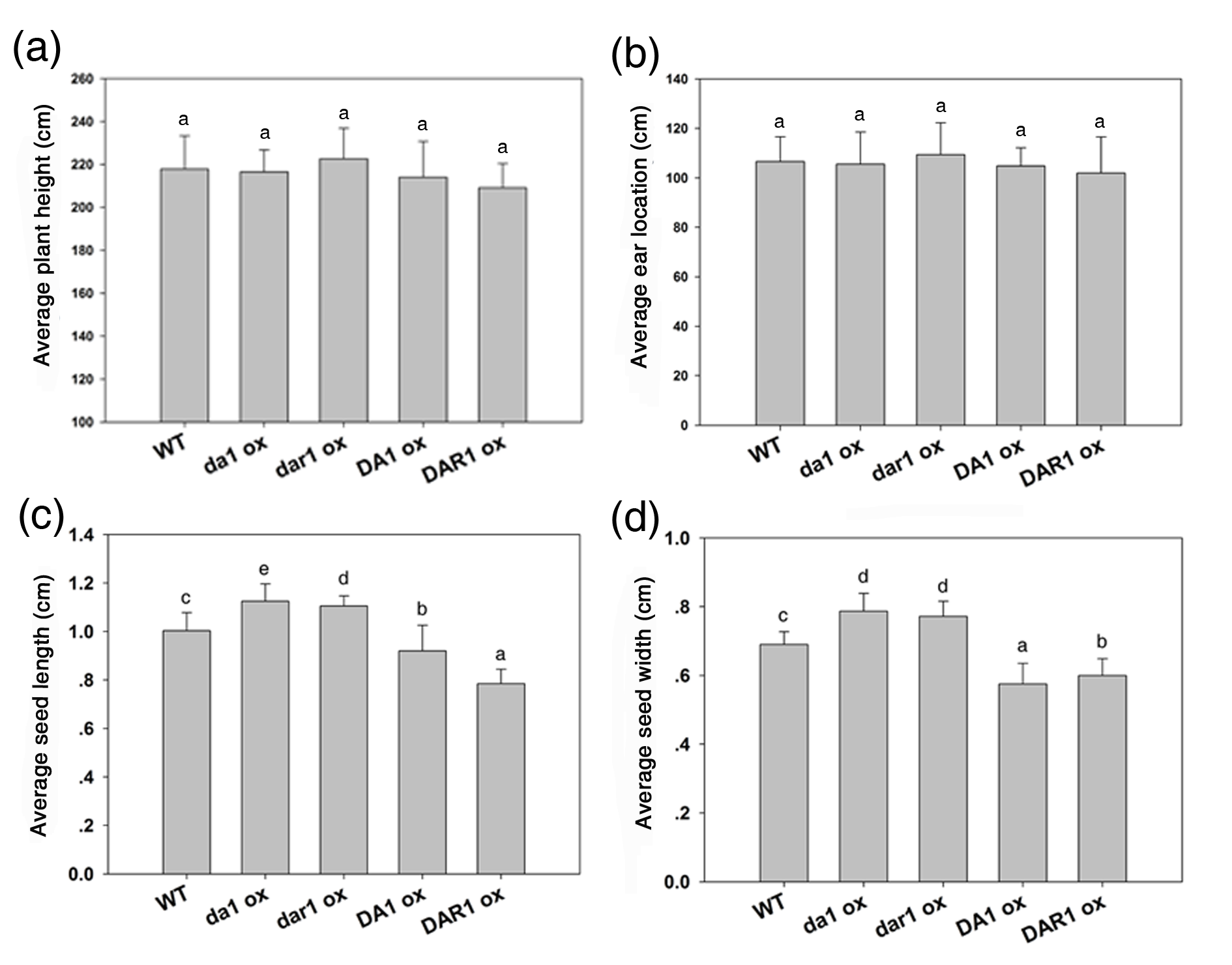

Supplement: Supplementary file 5 — Figure S5 The agronomic traits of the wild‐type and transgenic plants. [file PBI-16-234-s001.tif]
