# Supplementary material for: Over‐expression of mutated ZmDA1 or ZmDAR1 gene improves maize kernel yield by enhancing starch synthesis
Source: Plant Biotechnol J. 2017 Jul 25;16(1):234–44. doi: 10.1111/pbi.12763 (PMC5785342; doi:10.1111/pbi.12763)
Supplement: Supplementary file 7 — Table S2 Agronomic traits of WT and transgenic maize in the field in 2013 and 2015. [file PBI-16-234-s003.doc]

**Table S2 Agronomic traits of WT and transgenic maize in the field in 2013 and 2015**

| Year |  | Line | Ear length (cm) | Kernel number per row | Kernel number per ear | 100-grain number (g) | Grain weight per ear(g) | Grain weight per plot (Kg) | Row number per ear |
| --- | --- | --- | --- | --- | --- | --- | --- | --- | --- |
| 2013 |  | WT | 14.06 ±0.19 d | 31.00 ±2.00 c | 442.83 ±11.45 e | 23.80 ±0.24 f | 105.40 ±2.97 e | 5.06 ±0.14 e | 14.67 ±0.94 a |
|  | *da1* ox | La19 | 15.04 ±0.35 ef | 33.67 ±1.60 de | 499.83 ±9.39 g | 24.38 ±0.24 g | 121.86 ±1.76 g | 5.85 ±0.09 g | 15.00 ±1.53 a |
|  |  | La29 | 15.10 ±0.13 ef | 32.00 ±2.00 cd | 445.33 ±11.61 e | 27.53 ±0.27 j | 122.62 ±3.51 g | 5.89 ±0.17 g | 14.67 ±1.49 a |
|  |  | La30 | 15.32 ±0.29 fg | 32.67 ±1.25 cde | 470.67 ±12.61 f | 24.87 ±0.27 h | 117.04 ±3.28 f | 5.62 ±0.16 f | 14.33 ±0.75 a |
|  | *DA1* ox | LA12 | 11.45 ±0.41 b | 24.50 ±1.50 ab | 348.33 ±11.93 a | 18.53 ±0.29 b | 64.57 ±2.81 a | 3.10 ±0.14 a | 14.33 ±0.75 a |
|  |  | LA17 | 11.86 ±0.42 bc | 25.33 ±1.37 ab | 365.00 ±11.97 ab | 19.35 ±0.30 c | 70.61 ±2.16 b | 3.39 ±0.10 b | 14.00 ±0.00 a |
|  |  | LA23 | 10.83 ±0.41 a | 23.50 ±1.89 a | 361.00 ±22.91 ab | 18.20 ±0.31 a | 65.73 ±4.74 a | 3.15 ±0.23 a | 14.33 ±0.75 a |
|  | *dar1* ox | Lr38 | 14.70 ±0.22 e | 33.67 ±1.80 de | 504.50 ±10.24 g | 24.72 ±0.27 h | 124.70 ±3.17 gh | 5.99 ±0.15 gh | 15.33 ±0.94 a |
|  |  | Lr44 | 15.54 ±0.25 g | 34.50 ±1.50 e | 500.83 ±5.30 g | 25.80 ±0.24 i | 129.20 ±0.57 i | 6.20 ±0.03 i | 14.67 ±0.94 a |
|  |  | Lr62 | 14.88 ±0.40 e | 35.00 ±1.15 e | 497.67 ±6.29 g | 25.65 ±0.28 i | 127.65 ±2.28 hi | 6.13 ±0.11 hi | 14.00 ±0.00 a |
|  | *DAR1* OX | LR1 | 12.18 ±0.27 c | 26.50 ±2.06 b | 390.67 ±16.30 d | 20.52 ±0.16 e | 80.15 ±3.32 d | 3.85 ±0.16 d | 14.67 ±0.94 a |
|  |  | LR4 | 11.66 ±0.18 b | 25.67 ±1.60 ab | 378.00 ±14.19 cd | 19.75 ±0.27 d | 74.65 ±2.86 c | 3.58 ±0.14 c | 14.00 ±0.00 a |
|  |  | LR11 | 11.48 ±0.33 b | 25.50 ±1.71 ab | 368.83 ±15.59 b | 19.53 ±0.25 cd | 72.16 ±3.07 bc | 3.46 ±0.15 bc | 14.00 ±1.15 a |
|  |  |  |  |  |  |  |  |  |  |
| 2015 |  | WT | 13.52 ±0.19 d | 29.60 ±0.73 d | 435.23 ±13.20 d | 25.72 ±0.25 e | 107.16 ±1.55 d | 4.84 ±0.07 d | 15.60 ±0.73 bc |
|  | *da1* ox | La19 | 14.70 ±0.53 ef | 33.00 ±1.73 fg | 480.56 ±30.57 ef | 28.86 ±0.41 e | 138.70 ±4.83 e | 6.24 ±0.23 e | 15.33 ±0.94 ab |
|  |  | La29 | 14.54 ±0.18 ef | 31.20 ±1.06 e | 430.91 ±20.73 d | 29.86 ±0.35 g | 124.78 ±4.14 e | 5.51 ±0.20 e | 15.00 ±1.00 ab |
|  |  | La30 | 14.44 ±0.28 fg | 32.00 ±1.63 ef | 475.10 ±28.93 e | 27.80 ±0.12 f | 132.30 ±4.44 e | 5.95 ±0.21 e | 15.67 ±1.37 bc |
|  | *DA1* ox | LA12 | 10.55 ±0.19 b | 23.67 ±0.75 ab | 344.79 ±15.50 a | 21.77 ±0.33 c | 75.04 ±3.30 ab | 3.60 ±0.16 ab | 14.80 ±0.89 ab |
|  |  | LA17 | 11.03 ±0.18 bc | 24.67 ±0.75 abc | 354.56 ±11.65 ab | 21.18 ±0.29 ab | 75.12 ±2.96 ab | 3.61 ±0.14 ab | 14.40 ±0.73 ab |
|  |  | LA23 | 10.40 ±0.21 a | 23.17 ±0.69 a | 337.92 ±13.18 a | 20.68 ±0.31 a | 69.89 ±2.82 a | 3.35 ±0.14 a | 14.80 ±0.89 ab |
|  | *dar1* ox | Lr38 | 14.03 ±0.23 e | 33.40 ±0.73 fgh | 501.79 ±20.38 fg | 26.16 ±0.53 e | 116.92 ±7.65 e | 5.61 ±0.37 e | 16.67 ±0.94 c |
|  |  | Lr44 | 14.82 ±0.33 g | 34.60 ±1.69 h | 506.15 ±20.92 g | 26.92 ±0.09 f | 126.60 ±4.77 f | 6.08 ±0.23 f | 15.67 ±0.75 bc |
|  |  | Lr62 | 14.90 ±0.30 f | 34.40 ±1.59 gf | 506.34 ±20.41 g | 27.20 ±0.20 f | 128.70 ±6.54 f | 6.18 ±0.31 f | 15.67 ±0.75 bc |
|  | *DAR1* OX | LR1 | 11.70 ±0.22 c | 25.83 ±0.90 c | 378.51 ±9.95 c | 22.50 ±0.32 d | 85.17 ±2.81 c | 4.09 ±0.13 c | 14.80 ±0.89 ab |
|  |  | LR4 | 11.23 ±0.14 b | 25.00 ±0.58 bc | 360.62 ±9.58 ab | 21.90 ±1.04 c | 79.00 ±4.69 b | 3.79 ±0.23 b | 14.40 ±0.73 ab |
|  |  | LR11 | 11.20 ±0.25 b | 24.50 ±0.96 abc | 350.16 ±11.63 a | 21.42 ±0.56 bc | 75.05 ±4.33 ab | 3.60 ±0.21 ab | 14.00 ±0.00 a |

Values are mean ± SD and labeled with letter are significantly different at P<0.05 by Duncan’s test. n=18.
