# Supplementary material for: Over‐expression of mutated ZmDA1 or ZmDAR1 gene improves maize kernel yield by enhancing starch synthesis
Source: Plant Biotechnol J. 2017 Jul 25;16(1):234–44. doi: 10.1111/pbi.12763 (PMC5785342; doi:10.1111/pbi.12763)
Supplement: Supplementary file 8 — Table S3 The primer pairs used in this study. [file PBI-16-234-s004.doc]

| **Table S3 The primer pairs used in this study** | | |
| --- | --- | --- |
| **primer pairs for gene cloning and bridge PCR (from 5'to 3')** | |  |
| *ZmDA1* | 5'-CGGGGTACCATGGGTTGGCTGACCAAG-3' | 5'-CCGCTCGAGTCAAAATGGAAAGGTCCCAG-3' |
| m*ZmDA1* | 5'-CATCTTCCTGAAACAAGAGGAC-3' | 5'-GTCCTCTTGTTTCAGGAAGATG-3' |
| *ZmDAR1* | 5'-CGGGGTACCATGAGTTGGTTAAACAAAATTTTTAAGG-3' | 5'-CCGCTCGAGTTAATATGGGAAAGACCCTGTCAAC-3' |
| m*ZmDAR1* | 5'-CACCTACCGGAAACCAGAGGTC-3' | 5'-GACCTCTGGTTTCCGGTAGGTG-3' |
| **primers for bar gene detection (from 5'to 3')** | |  |
| bar | 5'-GCAGGAACCGCAGGAGTGGA-3' | 5'-AGCCCGATGACAGCGACCAC-3' |
| **primer pairs for probe in southern blot (from 5'to 3')** | |  |
| Bar SB | 5’-GCAGGAACCGCAGGAGTGGA-3' | 5’-AGCCCGATGACAGCGACCAC-3' |
| **primer pairs uesd in the qRT-PCR and semi-RT PCR (from 5'to 3')** | |  |
| qZmDA1 | 5'-ACTATGTATCATCCGCCTCG-3' | 5'-GGGTGAAACTGCTCCTTGTAG-3' |
| qZmDAR1 | 5'-ATTGGCCATGGACGTTTCC-3' | 5'-CCAGAAAGGATGTGCTCGG-3' |
| qZmACTIN1 | 5'-ATCACCATTGGGTCAGAAAGG-3' | 5'-GTGCTGAGAGAAGCCAAAATAGAG-3' |
| qZmINCW2 | 5'-GGAGCGTCGCATGTCGTCTAC-3' | 5'-CGCCCTTGGGATTGTATTGG-3' |
| qZmSWEET4c | 5'-GTGGCGACGCTGCTGAACTG-3' | 5'-ACGGAGACGATGCCGACGAG-3' |
| qZmBETL4 | 5'-ATGCGAAGAGTACATCAAGCCTG-3' | 5'-TGTCTGTCTACACCACCACCTTG-3' |
| qZmCYC2;3 | 5'-ACCAGTGCCAGAATAACAAG-3' | 5'-AACAGCCAGAGGGAGAAG-3' |
| qZmRBR2 | 5'-GGGAGCCTGAATCATCTA-3' | 5'-ACTTCTTCTATTGCCTTGTG-3' |
| qZmMRP1 | 5'-CGTAGTGCCATTGTGAACC-3' | 5'-CACTGGTGTCCTTGTGGG-3' |
| qZmBETL1 | 5'-TGTTGCCATTCTGTCCTCACT-3' | 5'-ACACCAGCCACCGTCCTT-3' |
| qZmTCRR1 | 5'-CCCGTGTTGATTGCCTTGT-3' | 5'-TGGCGATTCCTTCACTTCC-3' |
| qZmGBSSI | 5'-TACGAGACGGTCAGGTTCTTCC-3' | 5'-CCAGTCGTTGCAGACGAACAC-3' |
| qZmSh2 | 5'-TGTGAGGGTGATGGGATTG-3' | 5'-CGCCCAAAATGATAGCAGA-3' |
| qZmBrittle2 | 5'-GAAGCCAATACCAGATTTCAGC-3' | 5'-TGTCTCATAGTAGTCCGCACCC -3' |
| qZmSEBIIa | 5'-TGCTGCCAGATGTTGCCC-3' | 5'-CACAGCAGTACGGAGTATAGCGT-3' |
| qZmSBEIIb | 5'-CTCCAGTGGAGTGATAGCGG-3' | 5'-GGAAAGCGAAAAACGAAAAC-3' |
| qZmSBEI | 5'-GAAAGCAGAGACAGGAAAGACGT-3' | 5'-TGCAAGCTACAGGAAAGGACC-3' |
| *qZmSSI* | 5'-ACATTACATGGAAGGCAGACC-3' | 5'-CCTAACGAGCAAAGGACAAC-3' |
| *qZmSSIIa* | 5'-CAGTTTGCTGGAACCTTGT-3' | 5'-GTTGAGCCTGTTCTTAGCC-3' |
| *qZmSSIIIa* | 5'-CGGGCATGTATGTTTGTTT-3' | 5'-ACTCTACGGGCTAGTTTGG-3' |
